# Biomaterials and tissue engineering for scar management in wound care

**DOI:** 10.1186/s41038-017-0069-9

**Published:** 2017-01-21

**Authors:** Maedeh Rahimnejad, Soroosh Derakhshanfar, Wen Zhong

**Affiliations:** University of Manitoba, Winnipeg, MB Canada

**Keywords:** Biomaterials, Scar management, Wound healing, Tissue engineering

## Abstract

Scars are a natural and unavoidable result from most wound repair procedures and the body’s physiological healing response. However, they scars can cause considerable functional impairment and emotional and social distress. There are different forms of treatments that have been adopted to manage or eliminate scar formation. This review covers the latest research in the past decade on using either natural agents or synthetic biomaterials in treatments for scar reduction.

## Background

Scar tissue forms in injured areas of human bodies and replaces cells that have been destroyed. It appears either inside the body or on the skin. Scar tissue on the skin looks different from the surrounding area; while inside the body, scar may cause adhesion between tissues and organs or fibrosis. Scar causes functional impairment and emotional distress, thus the pre- or post-scar managements are important [1].

A scar is caused by the overgrowth of a tissue after an injury, burn, or surgical incision, demonstrating an exuberant healing response which determines type of scars: hypertrophic and keloid. Hypertrophic scars do not extend beyond the wound borders while keloid scars do. Hypertrophic scars are more favorable than the keloid scars clinically as they are more manageable in treatment and are often more spontaneous in regression. In a keloid scar, thicker and more irregularly arranged collagen often with pain is observed. For a hypertrophic scar, however, patients encounter thinner and more parallel arranged collagen in scars. Moreover, hypertrophic scars arise in all races with low probability in young and aged people [1]. Keloid scars, on the other hand, more often occur in non-white persons [2]. Cutaneous scars have attracted more research work than the others from the cosmetic perspective [3].

Wound healing is an intricate and dynamic process of devitalized and damaged cellular constructs and tissue layers replacement that normally occurs through the scar tissue formation. Wound healing process of a human adult can be classified into three distinct phases: (1) inflammatory, in which damaged and dead cells, along with pathogens or debris, are cleared out via the phagocytosis. Platelet-derived growth factors are released that cause the cells migration and division during the proliferation; (2) proliferation or new tissue formation and angiogenesis, collagen regeneration, growth of granulated tissue, epithelialization, and wound contraction occur; (3) remodeling stage, in which collagen is orientated along tension lines, and non-viable cells are removed by apoptosis. A number of growth factors and cytokines have been reported to be involved in the wound healing procedure through different biochemical pathways [4].

Scar formation is a prevalent, undesirable consequence of most wound healing events, along with significant psychological, emotional, and social problems [4–6]. It is always desirable but difficult to develop wound treatment that allows prompt healing and less scarring, particularly in adult tissues. Accordingly, there have been considerable research and development efforts to not only accelerate the healing process but also prevent the scar or minimize the scar size in skin or other tissues [7, 8].

In addition, many different techniques to treat scars have been developed, including laser therapy, diverse types of sutures, and radiation therapy. However, fewer methods have been surveyed for cutaneous scar prevention. For instances, Barbed sutures as a self-anchoring and knotless option were evaluated in surgery clinically and were shown to provide comparable performance and safety as compared to conventional wound closure techniques [9]. Spencer suggested that applying of pulsed-dye laser with the immune response modifier (IRM) imiquimod as a topical agent on surgery scar areas may help reduce scar size [10]. Moreover, Tsao et al. developed a tissue-sealing technology, photo-activated tissue bonding (PTB), which did sealing on a molecular scale [11]. Sobanko and Alster analyzed several laser systems on different facial cutaneous scars. Lasers reduce the depth of the scar borders and provide neocollagenesis, therefore improving skin irregularities [12]. Jiang et al. created a dopamine-based crosslinker-conjugated gelatin/polycaprolactone nanofibrous sheet to eliminate suture use in surgery which showed potentials to repair tissue and avoid suture-caused stress concentration [13]. To this end, we review the research on scar management in the past decades based on a variety of methods including pharmaceutical products, biomaterial-based dressings, cell therapy, and tissue engineering substitutes. Although there have been numerous works on biomaterials and tissue engineering for wound care, we will only discuss those that deal with scar management.

### Pharmaceutical products for scar management

Numerous documents have been found on wound treatments, but not much of them considered a scarless healing or scar minimization [14]. Pharmaceutical products including both traditional plant-based materials and proteins have been reported as effective wound cures to reduce or eliminate scars. This section is to provide a discussion on the pharmaceutical products that has been found to play a role in promoting wound healing and scar management (Table 1). However, it should be noted that these products, in practice, have to be used in combination with dressing or scaffolding biomaterials in wound care, as will be discussed in the next section.

**Table 1:** Pharmaceutical products for scar management

	Materials	In vivo/in vitro	Function	Ref
Pharmaceutical products for scar management	Pycnogenol	In vivo	Decreasing oxidized ascorbate, providing inhibitory effect on matrix metalloproteinases, and supporting collagen matrix formation	[15]
	Relaxin	In vivo	Enhancing the normal wound healing process by increasing angiogenesis, reducing scar formation and granulation tissue, and contributing to a well-organized collagen framework	[16]
	*Astragalus membranaceus* (AR)	In vivo	Suppressing inflammation and promoting basal cell proliferation, angiogenesis, and linear alignment of the granulation tissue	[17]
	Astragaloside IV	In vitro and in vivo	Inhibiting the transforming growth factor beta 1 (TGF-β1) secretion, regulating the ratio of collagen type I/type III in the remodeling stage to reduce scarring	[18]
	Crocodile oil	In vivo	Decreasing the messenger ribonucleic acid (mRNA) expressions of TGF-β1and Smad3	[19]
	Curcumin	In vivo	Suppressing TGF-β1/SMAD pathway and extra cellular matrix (ECM) production in primary keloid fibroblasts and reducing pro-inflammatory cytokines, interleukins (IL-1β, IL-6, and IL-8)	[20]
	Honey	In vitro	Stimulating monocytes (MM6 cells) to secrete cytokines, tumor necrosis factor-alpha (TNF-α), IL-1 and IL-6, degrading collagen IV through stimulation of the matrix metalloproteinases 9 (MMP-9) during the reepithelialization process of wound repair	[21]
	c-Ski	In vivo	Modulating wound healing and scar formation through modulating fibroblast functions, reducing scar formation by decreasing collagen production, and reducing the protuberant height and volume of scars and increasing collagen maturity in a hypertrophic scar model, effecting TGF-β1 signaling through both Smad2/3-dependent and Smad-independent pathways	[27, 28]
	Jun amino-terminal kinases (JNK)	In vivo	Mediating connective tissue growth factor expression in corneal wound healing	[29]
	Calpains	In vivo and in vitro	Playing a major role in granulation tissue formation	[30]
	MG53	In vivo and in vitro	Facilitating injury repair and inhibiting myofibroblast differentiation and an effective means for promoting scarless wound healing	[31]

*AR* astragalus membranaceus, *TGF-β1* transforming growth factor beta 1, *mRNA* messenger ribonucleic acid,* ECM* extracellular matrix, *IL* interleukins, *TNF-α* tumor necrosis factor alpha, *MMP-9* matrix metalloproteinases 9, *JNK* jun amino-terminal kinases

Naturally derived materials have long been used as a substantial source of medicines. Natural materials are still considered as ideal sources for a broad range of diseases. Pycnogenol, an extract from French maritime pine bark containing a mixture of procyanidins, was reported as a wound healing accelerator and scar formation reducer. Pycnogenol was suspended in a polyacrylic acid hydrogel and tested in vivo. Pycnogenol was found to decrease oxidized ascorbate and, consequently, to provide inhibitory effect on matrix metalloproteinases and to support collagen matrix formation. [15].

Relaxin was tested on rodent wound healing: Juvenile pigs were used for an investigation of scar reduction and cosmetic outlook because of their similar healing mechanism to human. It was demonstrated that wounds treated with relaxin have less granulation and inflammation, and more well-knit collagen framework, representing that relaxin boosts the normal wound repair procedure by increasing angiogenesis, reducing scar formation and granulation tissue, and contributing to a well-organized collagen structure [16].

The effect of natural materials on decreasing the scar size has been illustrated in many studies. Han and colleagues reported that topically applied extract from the root of *Astragalus membranaceus* (AR) incorporated in a hydrophilic foam dressing is effective in increasing the closure of rats’ acute open wounds. AR was found to suppress inflammation and promote basal cell proliferation, angiogenesis, and linear alignment of the granulation tissue and, consequently, to result in faster wound healing procedure [17].

Chen et al. further revealed the healing and anti-scar effects of astragaloside IV on the wound cure improvement in vitro and in vivo. It was reported that astragaloside IV can inhibit the transforming growth factor beta 1 (TGF-β1) secretion and improve healing. In addition, it can regulate collagen type I/type III ratio in the remodeling phase to reduce scarring [18].

Li et al. examined the effect of crocodile oil in enhancing wound healing process and decreasing scar formation in rats. They found that crocodile oil significantly decreased the messenger ribonucleic acid (mRNA) expressions of TGF-β1and Smad3, which are the key cytokines that play a role in accelerated wound healing and less scar formation [19].

Another natural therapeutic agent which has been found to have anti-inflammatory and antioxidant characteristics, curcumin, was recently tested on rabbit ear wounds, suggesting that the systemic administration of curcumin improves lesion repair and reduces scarring. Curcumin was found to suppress TGF-β1/SMAD pathway and extra cellular matrix (ECM) production in primary keloid fibroblasts and reduce pro-inflammatory cytokines, interleukins (IL-1β, IL-6, and IL-8), which directly decrease hypertrophic scarring [20].

Honey, a traditional medical ingredient known for thousands of years, was reviewed for its healing and anti-microbial capacities. Honey stimulates monocytes (MM6 cells) to secrete cytokines, tumor necrosis factor alpha (TNF-α) and IL-1 and IL-6, which triggers the immune reaction to infection. Honey helps collagen IV degradation via the matrix metalloproteinases 9 (MMP-9) stimulation during the reepithelialization phase of wound healing [21]. Previous research showed that honey has wound therapeutic properties including the enhancement of autolytic debridement, growth of wound tissues, and anti-inflammatory activities. Honey has therefore been suggested to provide better wound repair and advance scarring processes. Honey is found to be more efficient and beneficial in eliminating microbial contamination, reducing scar formation, and promoting epithelial regeneration in comparison with other topical agents like silver nanoparticles [22–26].

On the other hand, some scientists are interested in modulating and stimulating wound healing signal pathways by using bioactive substitute such as proteins, enzymes, or growth factors. c-Ski is a tissue repair-related gene that is mostly expressed in fibroblasts during the cell proliferation stage of wound healing; Liu et al. suggested that c-ski is capable of controlling scarring in wound repair by modulating fibroblast functions. They studied the effects of c-Ski on skin fibroblast proliferation, collagen type I secretion, and myofibroblast differentiation [27]. The same group later also showed the potential of c-Ski in scar reduction by suppressing the production of protein in rat cutaneous wounds, as well as the effect of c-Ski in reducing scar size in a hypertrophic scar in a rabbit ear model. It effects TGF-β1 signaling via Smad2/3-dependent and Smad-independent pathways that minimize scar formation and speed up wound healing [28].

Shi et al. worked on Jun amino-terminal kinases (JNK) signaling to mediate healing of corneal wound via expression of connective tissue growth factor, thus demonstrated that JNK can potentially serve as a new strategy to help in corneal scar reduction [29]. The effect of calpains as cellular adhesion, motility, and inflammation and angiogenesis regulator protease was surveyed on scar formation. The results suggested that calpains play a major role in granulation tissue formation. An inhibition of calpains, therefore, should be considered for treatments aiming at scar reduction [30].

Recently, wound healing and scarring was studied from a membrane repair gene, MG53 point of view. The study established that MG53 can be a facilitator of injury repair and inhibitor of myofibroblast differentiation and an effective means for promoting scarless wound healing [31].

Generally speaking, the pharmaceutical products studied for their effects on promoting wound healing and scar formation do not have strong evidence to support their efficacy. On the one hand, more studies are necessary to examine their efficacy and biochemical mechanism in terms of the roles they played in wound healing and scar management. On the other hand, these pharmaceutical products have to be used in combination with dressing or scaffolding biomaterials in wound care, which will be discussed in the next section.

### Biomaterial based dressings for scar management

**Fig. 1:**
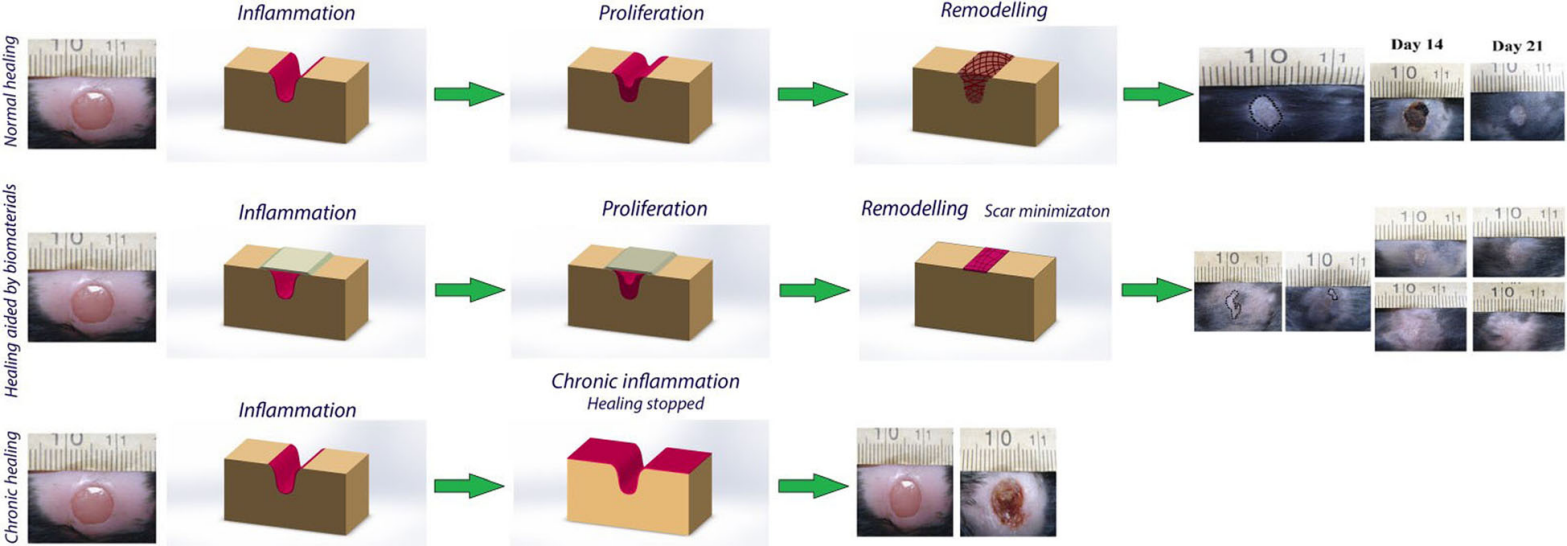
The efficacy of cell-laden biomaterial-based dressing in wound healing for scar management [74]

To improve the traditional scar management, new advanced wound dressings have been developed to increase wound healing capacity. The biomaterials composed of natural polymers and bioactive molecules briefly summarized in Table 2. The healing progression and scar reduction by biomaterial-based dressings have been shown as schematic in Fig. 1.

**Table 2:** Biomaterial-based dressings for scar management

	Materials	In vivo/vitro	Function	Ref
Biomaterials composed of natural polymers	Hyaluronic acid (HA) in ECM	In vivo	Reducing TGF-β1 level in the wound, maintaining optimal viscoelastic properties of the ECM, and decreasing levels of fibronectin, fibromodulin, procollagen I, and HA synthase	[32–34]
	Genipin cross-linked gelatin (GCG) and collagen sheets	In vivo	Scarless nerve regeneration, favorable nerve functional recovery	[35]
	Microbial cellulose	In vivo	Improving the healing rate, decreasing pain and reducing scar tissue formation, necrotic debris removal, new cell migration and growth, and prompting reepithelialization	36
	Collagen membrane cross-linked with glutaraldehyde	In vivo	Oral scar reducing, controlling infection in primary healing stage, and reducing growth of granulation tissue	[37]
	Electrospun nanofibrous dressings composited of silk fibroin/gelatin and cellulose acetate	In vivo and in vitro	Increasing expression of vascular endothelial growth factor (VEGF) and existence of collagen type I	[38, 39]
	Electrospun silk fibroin nanomatrix	In vivo	Reducing the wound healing period and scar formation	[40]
Biomaterials incorporated with bioactive molecules	Genipin-modified collagen sheets	In vivo	Reducing scars in first- and second-degree burns, assisting the synthesis of neodermal collagen matrices	[35, 41]
	Polyvinyl alcohol–sodium alginate gel-matrix-based wound dressing system containing nitrofurazone	In vivo	Keeping wound moist and prevent secondary damage, mild positive effects on inflammatory phase and create reducing wound size	[42]
	Multifunctional acellular biologic scaffold	In vivo	Selective delivery and release of shielded biomaterials and bioactive substances, scaffolds help in vascularization, blood vessel formation, and keeps body temperature	[43]
	Commercial calcium alginate	In vivo	Scarring prevention by moisture management and regulating amount of exudates in wound during the healing	[45]
	Ginsenoside Rg3-loaded electrospun poly(lactic-co-glycolic acid) (PLGA) fibrous membranes	In vitro	Scar prevention, decreasing the expression of vascular endothelial growth factor (VEGF), mRNA, and collagen type I	[49]
	Norfloxacin-loaded collagen/chitosan scaffold	In vivo	Controlling infection which contributes to lower inflammation, higher new cell growth, and faster wound closure	[50]
	Dressing of polyester fabric containing elemental silver and zinc	In vivo	Promoting collagen synthesis and reepithelialization rate	[51, 52]

*HA* hyaluronic acid, *ECM* extracellular matrix, *TGF-β1* transforming growth factor beta 1, *GCG* genipin cross-linked gelatin, *VEGF* vascular endothelial growth factor, *PLGA* poly(lactic-co-gly acid)

#### Biomaterials composed of natural polymers

A number of biopolymers derived from natural resources have been used in wound care for reducing scar formation. As described by Hu et al., fetal tissue heals rapidly without scarring due to the higher levels of hyaluronic acid (HA) in ECM as compared to adult tissues. Therefore, they observed that HA strand grafts enhanced wound closure rate and reduced the scar area remarkably by reducing TGF-β1 level in the wound [32]. Injectable HA hydrogels were also used to improve wound healing and scarring, resulting in remarkably less fibrosis than wounds without injection in a rabbit model. HA was found to maximize healing and minimize scar formation through preserving optimal viscoelastic properties of the ECM [33]. Another study was accomplished on the efficiency of HA hydrogels in treatment of scarring on rabbits. The results of this study demonstrated that a prophylactic use of a chemically modified HA hydrogel may increase the wound cure feature of HA in regenerating tissues by decreasing levels of fibronectin, fibromodulin, TGF-β1, procollagen I, and HA synthase and improving wound viscoelastic properties [34].

Genipin cross-linked gelatin (GCG) and collagen sheets were analyzed on minimizing invasion and scarring of the nerve and open wound healing in vivo. It was reported that GCG can be a beneficial aid for scarless nerve regeneration and lead to desirable nerve functional recovery [35].

Czaja et al. studied microbial cellulose and its effect on patients with second-degree facial burn. Compared to a standard technique with moist gauze dressing and ointment, the dressing considerably promoted the healing rate in deep facial burns. A decrease of pain and reduction of scar tissue formation were also observed for wounds treated by the microbial cellulose dressing. The moist environment created using the dressing facilitates necrotic debris removal, new cell migration and growth, and prompted reepithelialization [36].

Another trial was done on collagen membrane cross-linked with glutaraldehyde as a wound dressing for surgical defects of the oral mucosa. Based on the study, it was an excellent wound graft material for oral scar reducing; however, there was some contraction and moderate scarring in some patients who lost their collagen membrane early. This graft was reported to control infection in primary healing stage and to reduce growth of granulation tissue, leading to less scarring and short healing duration [37].

Electrospun nanofibrous dressings composited of silk fibroin/gelatin and cellulose acetate have been examined in vivo and in vitro to prove their functionality in mimicking skin regeneration and in reducing scar formation [38, 39]. Wounds covered with the nanofibrous dressings showed increased expression of VEGF and existence of collagen type I which is similar to the normal skin [38]. Electrospun silk fibroin nanomatrix fabricated as wound dressing materials were recently evaluated for burn wound repair as compared to clinically used dressings. Such an electrospun nanomatrix was found to reduce the wound healing period and scar formation. Amount of some involved growth factor and cytokines such as TGF-β1, IL-1α, 6, and 10 evaluated and ascertained their regulation that recovers epidermis [40].

#### Biomaterials incorporated with bioactive molecules

Recently, there have also been work that employed wound dressings incorporated with drugs or bioactive molecules to enhance their capacity in scar management. It was demonstrated that the genipin-modified collagen sheets are effective in reducing scars in first- and second-degree burns [35, 41]. Collagen sheets were reported to assist in the synthesis of neodermal collagen matrices for scarless healing [41].

Kim et al. developed a polyvinyl alcohol–sodium alginate gel-matrix-based wound dressing structure containing nitrofurazone, which performs a positive healing and less scarring effect as compared with wound dressing without nitrofurazone. The drug-loaded hydrogels, as a biodegradable and nontoxic polymeric matrix, keep wound moist and prevent secondary damage when dressings are changed. They show mild positive effects on inflammatory phase and create reduced wound size with new epithelium noted at the edge of the defects [42].

A multifunctional acellular biologic scaffold was combined with wound dressings which has selective delivery and release of shielded biomaterials and bioactive substances. It can be used in wounds or damaged tissues for scarolysis and eliminating dead debris. It was suggested that these scaffolds help in vascularization, blood vessel formation and keep body temperature [43].

In addition, Morton and Philips investigated a wound dressing which decreases scar formation by keeping moisture in the wound [44]. Some commercial calcium alginate dressing was also found to exhibit a capacity of scarring prevention by moisture management and regulating amount of exudates in wound during the healing [45].

Other groups reported efficiency of electrospun nanofibers and hydrogels for the treatment of diabetic ulcers. Particularly, nanofibrous meshes immobilized with basic fibroblast growth factor (bFGF) and epidermal growth factor (EGF), and dressings composed of polyvinylpyrrolidone (PVP), polyethylene glycol (PEG), and agar were studied to reveal their efficacies in reducing scarring [46–48].

In the past year or two, more advanced approaches have been employed in the prevention and minimization of scars. Some in vitro biomaterial membranes studies specialized in scar prevention application such as ginsenoside Rg3-loaded electrospun poly(lactic-co-glycolic acid) (PLGA) fibrous membranes as cutaneous wound cover [49]. As a result of using the biomembranes, the expression of vascular endothelial growth factor (VEGF), mRNA and Collagen Type I was found to decrease significantly, and consequently, to cause shorter healing time and to inhibit hypertrophic scar formation [49].

In another study, Norfloxacin-loaded collagen/chitosan scaffold was found to enhance the rate of wound healing with unnoticeable inflammation and scarring by controlling infection which contribute to lower inflammation, higher new cell growth and faster wound closure [50].

Blount and Harding developed a bioelectric dressing and tested it in vivo, resulting in faster wound epithelization and improved scar appearance. Specifically, a single-layer dressing was constructed from a polyester fabric containing elemental silver and zinc microcells held in position by a biocompatible binder which produced microcurrent using conductive fluid. The created microcurrent promoted collagen synthesis and reepithelialization rate [51, 52].

There has also been a large amount of work involving the use of biomaterials incorporated with viable cells (including stem cells). Such tissue engineering approaches for scar management will be discussed in the next section.

### Cell therapy and tissue engineering substitute for scar management

Cell therapy combined with scaffolding biomaterials have been employed in tissue engineering approaches for wound care and scar management.

Zaulyanov and Kirsner reviewed Apligraf, a bi-layered bioengineered skin substitute, the first of its kind approved by the US Food and Drug Administration (FDA) for treating venous leg ulcers and diabetic foot ulcers. The graft is developed from neonatal cells and may stimulate a more fetal-like scarless wound healing, therefore may result in better cosmetic appearance [53].

In another research, clinical outcomes of cultured epithelial autografts were investigated on facial skin defects. Although the cell cultures of an epithelial autograft did not fully meet the demands of the patients, especially in the younger patients with facial skin defects, it did reduce and improve scarring. As a result of using the autograft, faster vascularization, epithelial cells proliferation and migration have been observed in wounds [54].

To date, there have been extensive studies on tissue engineering and stem cells application in wound treatment and scar decreasing. Dermal and epidermal substitutes have been developed to help facilitate reepithelialization but still fail to reconstruct the appearance of the skin to its pre-wounded state [55]. Cellular therapies can be delivered by 3-dimensional structures (tissue-engineered live cell structures) that can be put topically over wound and scar surfaces as creams or gels.

Scientific findings in wound and scar management were highlighted by developing biological treatments with fetal cell therapy [56–59]. Fetal cells are differentiable cells with high capacity of expansion, regeneration, and low immunogenic properties and therefore may induce scarless wound healing or minimal scarring [60–64]. Skin substitutes developed from neonatal or young foreskin tissue cultures were shown to close wounds completely and rapidly and to regenerate the tissues with minimal scarring. They promote epithelial cells and fibroblasts proliferation and migration changes [65]. Wulff et al. further investigated the roles of mast cells in the healing procedure and found that they may regulate the changes from scarless to fibrotic healing. In this study, they verified the hypothesis that in the absence of mast cells, scar formation can be controlled and reduced [66].

There has also report on the capacity of mesenchymal stem cells (MSCs) in attenuating scar formation during wound healing by promoting angiogenesis and modulating the inflammatory responses [67]. Williams et al. tried to minimize durable scar size in ischemic cardiomyopathy by injecting allogeneic MSCs, suggesting that MSCs may reverse ventricular remodeling through durable infarct size reduction. It was demonstrated that MSCs can simulate endogenous cardiac stem cells to proliferate and differentiate, and adult cardiomyocytes re-enter the cell cycle via secreting plenty of growth factors and cytokines [68]. Beside, artificial dermis consisted of type I collagen fiber coated with 3% a-elastin hydrolysate reduced human burn wound contracture and promoted dermal reconstruction [69]. In most recent reports, MSCs were encapsulated in and delivered by gelatin microsphere and gelatin microcryogels to the margins of dermal wound and were found to accelerate wound closure rate and prevent scarring by maintaining MSC-released protein [70, 71]. Li et al. demonstrated that 3-dimentional graphene foam (3D-GF) loaded with MSCs decreases scar formation, potentially because of biomechanical and biochemical signals from 3D-GFs. The foam provided upregulation of VEGF and bFGF to neovascularize, downregulation of TGF-β1 and alpha-smooth muscle actin (α-SMA) together with an enhancing of TGF-β3 to prevent scarring [72].

Polyhydroxybutyrate-co-hydroxyvalerate constructs loaded with adipose-derived stem cells (ASCs) were shown to maintain the wound moisture and assert appropriate mechanical properties to endure wound contraction. Furthermore, exudate and inflammatory cell infiltration were found to promote the structure degradation and, consequently, improve scarless repair. The scaffold was found to promote expression of VEGF and bFGF with the presence of ASCs for appropriate blood vessel formation and played an important role in scar formation management by regulation of TGF-β1, α-SMA, and TGF-β3 [73].

## Conclusions

In this review, we have covered the methods and technologies in scarless wound healing in the past decade. Extensive studies have been concentrated on treatment rather than prevention and minimizing of postsurgical and traumatic scars; however, here, we have discussed several investigated ways and wound managements which are more likely to provide better cosmetic outcomes by scar reduction. Generally, understanding different types of treatments in human wound healing process in order to perfectly regenerate their missing cell and tissue may propose strategies and methods for maximizing healing benefits and decreasing scarring. To attain these goals, future research should aim to identify critical factors in tissue repair and regeneration. It is plausible that this will pave the way forward in future developments for more advanced methods that will tackle the problems of scarring.

## Abbreviations

3D-GF: Three-dimentional graphene foam
AR: *Astragalus membranaceus*
ASCs: Adipose-derived stem cells
bFGF: Fibroblast growth factor
ECM: Extra cellular matrix
EGF: Epidermal growth factor
FDA: Food and Drug Administration
GCG: Genipin cross-linked gelatin
HA: Hyaluronic acid
IL: Interleukin
IRM: Immune response modifier
JNK: Jun amino-terminal kinases
MMP-9: Matrix metalloproteinases 9
mRNA: messenger ribonucleic acid
MSCs: Mesenchymal stem cells
PEG: Polyethylene glycol
PLGA: Poly(lactic-co-glycolic acid)
PTB: Photo-activated tissue bonding
PVP: Polyvinylpyrrolidone
TGF: Transforming growth factor
TNF-α: Tumor necrosis factor-alpha
VEGF: Vascular endothelial growth factor
α-SMA: Alpha-smooth muscle actin

## References

[1] Khunger N, Ganjoo A. Scar Management-ECAB: Elsevier Health Sciences; 2013.

[2] Grieb G, Steffens G, Pallua N, Bernhagen J, Bucala R (2011). Circulating fibrocytes–biology and mechanisms in wound healing and scar formation. Int Rev Cell Mol Biol.

[3] Wong JW, Gallant‐Behm C, Wiebe C, Mak K, Hart DA, Larjava H (2009). Wound healing in oral mucosa results in reduced scar formation as compared with skin: evidence from the red Duroc pig model and humans. Wound Repair Regen.

[4] Bielefeld KA, Amini-Nik S, Alman BA (2013). Cutaneous wound healing: recruiting developmental pathways for regeneration. Cell Mol Life Sci.

[5] Bloemen MC, van der Veer WM, Ulrich MM, van Zuijlen PP, Niessen FB, Middelkoop E (2009). Prevention and curative management of hypertrophic scar formation. Burns.

[6] Boateng J, Catanzano O (2015). Advanced Therapeutic Dressings for Effective Wound Healing—A Review. J Pharm Sci.

[7] Profyris C, Tziotzios C, Do Vale I (2012). Cutaneous scarring: Pathophysiology, molecular mechanisms, and scar reduction therapeutics: Part I. The molecular basis of scar formation. J Am Acad Dermatol.

[8] Tziotzios C, Profyris C, Sterling J (2012). Cutaneous scarring: pathophysiology, molecular mechanisms, and scar reduction therapeutics: part II. Strategies to reduce scar formation after dermatologic procedures. J Am Acad Dermatol.

[9] Murtha AP, Kaplan AL, Paglia MJ, Mills BB, Feldstein ML, Ruff GL (2006). Evaluation of a novel technique for wound closure using a barbed suture. Plast Reconstr Surg.

[10] Gorgos D (2006). New treatments minimize surgical scars. Dermatol Nurs.

[11] Tsao S, Yao M, Tsao H, Henry F, Zhao Y, Kochevar J (2012). Light‐activated tissue bonding for excisional wound closure: a split‐lesion clinical trial. Br J Dermatol.

[12] Sobanko JF, Alster TS (2011). Laser treatment for improvement and minimization of facial scars. Facial Plast Surg Clin North Am.

[13] Jiang J, Wan W, Ge L, Bu S, Zhong W, Xing M (2015). Mussel-inspired nanofibrous sheet for suture-less stomach incision surgery. Chem Commun.

[14] Budovsky A, Yarmolinsky L, Ben‐Shabat S (2015). Effect of medicinal plants on wound healing. Wound Repair Regen.

[15] Blazso G, Gabor M, Schönlau F, Rohdewald P (2004). Pycnogenol® accelerates wound healing and reduces scar formation. Phytother Res.

[16] Stewart DR (2009). Scar prevention and cosmetically enhanced wound healing using relaxin. Ann N Y Acad Sci.

[17] Han D, Lee H, Hahm D (2009). Wound-healing activity of Astragali Radix in rats. Methods Find Exp Clin Pharmacol.

[18] Chen X, Peng L-H, Li N, Li Q-M, Li P, Fung K-P (2012). The healing and anti-scar effects of astragaloside IV on the wound repair in vitro and in vivo. J Ethnopharmacol.

[19] Li HL, Chen LP, Hu YH, Qin Y, Liang G, Xiong YX (2012). Crocodile oil enhances cutaneous burn wound healing and reduces scar formation in rats. Acad Emerg Med.

[20] Jia S, Xie P, Hong SJ, Galiano R, Singer A, Clark RA (2014). Intravenous curcumin efficacy on healing and scar formation in rabbit ear wounds under nonischemic, ischemic, and ischemia–reperfusion conditions. Wound Repair Regen.

[21] Molan P, Rhodes T (2015). Honey: A Biologic Wound Dressing. Wounds.

[22] Zanier E, Bordoni B (2015). A multidisciplinary approach to scars: a narrative review. J Multidiscip Healthc.

[23] Jull AB, Cullum N, Dumville JC, Westby MJ, Deshpande S, Walker N. Honey as a topical treatment for wounds. Cochrane Libr. 2015. Cochrane Database Syst Rev. 2013;(2):CD005083. 10.1002/14651858.CD005083.pub4 PMC9719456 25742878

[24] Estevinho L, Pereira AP, Moreira L, Dias LG, Pereira E (2008). Antioxidant and antimicrobial effects of phenolic compounds extracts of Northeast Portugal honey. Food Chem Toxicol.

[25] Zohdi R, Mukhtar S, Said S, Azmi N, Ali A, editors. A comparative study of the wound healing properties of Gelam honey and silver sulfadiazine in diabetic rats. Biomedical Engineering and Sciences (IECBES), 2014 IEEE Conference on; 2014: IEEE.

[26] Yaghoobi R, Kazerouni A (2013). Evidence for clinical use of honey in wound healing as an anti-bacterial, anti-inflammatory anti-oxidant and anti-viral agent: A review. Jundishapur Journal Nat Pharm Prod.

[27] Liu X, Li P, Chen XY, Zhou YG (2010). c‐Ski promotes skin fibroblast proliferation but decreases type I collagen: implications for wound healing and scar formation. Clin Exp Dermatol.

[28] Li P, Liu P, Xiong RP, Chen XY, Zhao Y, Lu WP (2011). Ski, a modulator of wound healing and scar formation in the rat skin and rabbit ear. J Pathol.

[29] Shi L, Chang Y, Yang Y, Zhang Y, Yu F, Wu X (2012). Activation of JNK signaling mediates connective tissue growth factor expression and scar formation in corneal wound healing. PLoS One.

[30] Nassar D, Letavernier E, Baud L, Aractingi S, Khosrotehrani K (2012). Calpain activity is essential in skin wound healing and contributes to scar formation. PLoS One.

[31] Li H, Duann P, Lin P-H, Zhao L, Fan Z, Tan T (2015). Modulation of Wound Healing and Scar Formation by MG53 Protein-mediated Cell Membrane Repair. J Biol Chem.

[32] Hu M, Sabelman EE, Cao Y, Chang J, Hentz VR (2003). Three‐dimensional hyaluronic acid grafts promote healing and reduce scar formation in skin incision wounds. J Biomed Mater Res B Appl Biomater.

[33] Hansen JK, Thibeault SL, Walsh JF, Shu XZ, Prestwich GD (2005). In vivo engineering of the vocal fold extracellular matrix with injectable hyaluronic acid hydrogels: early effects on tissue repair and biomechanics in a rabbit model. Ann Otol Rhinol Laryngol.

[34] Thibeault SL, Klemuk SA, Chen X, Johnson BHQ (2011). In vivo engineering of the vocal fold ECM with injectable HA hydrogels—late effects on tissue repair and biomechanics in a rabbit model. J Voice.

[35] Chen Y-S, Chang J-Y, Cheng C-Y, Tsai F-J, Yao C-H, Liu B-S (2005). An in vivo evaluation of a biodegradable genipin-cross-linked gelatin peripheral nerve guide conduit material. Biomaterials.

[36] Czaja W, Krystynowicz A, Kawecki M, Wysota K, Sakiel S, Wróblewski P, et al. Biomedical applications of microbial cellulose in burn wound recovery. Cellulose: Molecular and structural biology: Springer; 2007. p. 307–21.

[37] Rastogi S, Modi M, Sathian B (2009). The efficacy of collagen membrane as a biodegradable wound dressing material for surgical defects of oral mucosa: a prospective study. J Oral Maxillofac Surg.

[38] Shan Y-H, Peng L-H, Liu X, Chen X, Xiong J, Gao J-Q (2015). Silk fibroin/gelatin electrospun nanofibrous dressing functionalized with astragaloside IV induces healing and anti-scar effects on burn wound. Int J Pharm.

[39] Atila D, Keskin D, Tezcaner A (2015). Cellulose acetate based 3-dimensional electrospun scaffolds for skin tissue engineering applications. Carbohydr Polym.

[40] Ju HW, Lee OJ, Lee JM, Moon BM, Park HJ, Park YR (2016). Wound healing effect of electrospun silk fibroin nanomatrix in burn-model. Int J Biol Macromol.

[41] Lazovic G, Colic M, Grubor M, Jovanovic M (2005). The application of collagen sheet in open wound healing. Ann Burns Fire Disasters.

[42] Kim JO, Park JK, Kim JH, Jin SG, Yong CS, Li DX (2008). Development of polyvinyl alcohol–sodium alginate gel-matrix-based wound dressing system containing nitrofurazone. Int J Pharm.

[43] Drago H, Marín G, Sturla F, Roque G, Mártire K, Aquino VD (2010). The next generation of burns treatment: intelligent films and matrix, controlled enzymatic debridement, and adult stem cells. Transplant Proc.

[44] Morton LM, Phillips TJ (2012). Wound healing update. Semin Cutan Med Surg.

[45] Kammerlander G, Eberlein T (2002). An assessment of the wound healing properties of Algisite M dressings. Nurs Times.

[46] Yang Y, Xia T, Chen F, Wei W, Liu C, He S (2011). Electrospun fibers with plasmid bFGF polyplex loadings promote skin wound healing in diabetic rats. Mol Pharm.

[47] Choi JS, Choi SH, Yoo HS (2011). Coaxial electrospun nanofibers for treatment of diabetic ulcers with binary release of multiple growth factors. J Mater Chem.

[48] Biazar E, Roveimiab Z, Shahhosseini G, Khataminezhad M, Zafari M, Majdi A. Biocompatibility evaluation of a new hydrogel dressing based on polyvinylpyrrolidone/polyethylene glycol. BioMed Res Int. 2011: 343989. 10.1155/2012/343989 PMC3155791 21860588

[49] Sun X, Cheng L, Zhu W, Hu C, Jin R, Sun B (2014). Use of ginsenoside Rg3-loaded electrospun PLGA fibrous membranes as wound cover induces healing and inhibits hypertrophic scar formation of the skin. Colloids Surf B: Biointerfaces.

[50] Mahmoud AA, Salama AH (2015). Norfloxacin-loaded collagen/chitosan scaffolds for skin reconstruction: Preparation, evaluation and in-vivo wound healing assessment. Eur J Pharm Sci.

[51] Blount AL, Foster S, Rapp DA, Wilcox R (2012). The use of bioelectric dressings in skin graft harvest sites: A prospective case series. J Burn Care Res.

[52] Harding AC, Gil J, Valdes J, Solis M, Davis SC (2012). Efficacy of a bio-electric dressing in healing deep, partial-thickness wounds using a porcine model. Ostomy Wound Manage.

[53] Zaulyanov L, Kirsner RS (2007). A review of a bi-layered living cell treatment (Apligraf®) in the treatment of venous leg ulcers and diabetic foot ulcers. Clin Interv Aging.

[54] Kim DM, Schwerdtner O, Schmidt-Westhausen A-M, Kage A, Klein M (2007). Cultured epithelial autografts in the treatment of facial skin defects: clinical outcome. J Oral Maxillofac Surg.

[55] Dieckmann C, Renner R, Milkova L, Simon JC (2010). Regenerative medicine in dermatology: biomaterials, tissue engineering, stem cells, gene transfer and beyond. Exp Dermatol.

[56] Hirt-Burri N, Ramelet A-A, Raffoul W, de Buys Roessingh A, Scaletta C, Pioletti D, et al. Biologicals and fetal cell therapy for wound and scar management. ISRN dermatology. 2011: 549870. 10.5402/2011/549870 PMC3262533 22363853

[57] Hasegawa T, Suga Y, Mizoguchi M, Ikeda S, Ogawa H, Kubo K (2004). Clinical trial of allogeneic cultured dermal substitute for the treatment of intractable skin ulcers in 3 patients with recessive dystrophic epidermolysis bullosa*. J Am Acad Dermatol.

[58] Mostow EN, Haraway GD, Dalsing M, Hodde JP, King D, Group OVUS (2005). Effectiveness of an extracellular matrix graft (OASIS Wound Matrix) in the treatment of chronic leg ulcers: a randomized clinical trial. J Vasc Surg.

[59] Shakespeare PG (2005). The role of skin substitutes in the treatment of burn injuries. Clin Dermatol.

[60] Hohlfeld J, de Buys Roessingh A, Hirt-Burri N, Chaubert P, Gerber S, Scaletta C (2005). Tissue engineered fetal skin constructs for paediatric burns. Lancet.

[61] De Buys R, Anthony S, Hohlfeld J, Scaletta C, Hirt-Burri N, Gerber S (2006). Development, characterization, and use of a fetal skin cell bank for tissue engineering in wound healing. Cell Transplant.

[62] Ramelet A-A, Hirt-Burri N, Raffoul W, Scaletta C, Pioletti DP, Offord E (2009). Chronic wound healing by fetal cell therapy may be explained by differential gene profiling observed in fetal versus old skin cells. Exp Gerontol.

[63] Applegate LA, Scaletta C, Hirt-Burri N, Raffoul W, Pioletti D (2009). Whole-cell bioprocessing of human fetal cells for tissue engineering of skin. Skin Pharmacol Physiol.

[64] Buchanan EP, Longaker MT, Lorenz HP (2009). Fetal skin wound healing. Adv Clin Chem.

[65] Fimiani M, Pianigiani E, Di Simplicio FC, Sbano P, Cuccia A, Pompella G (2005). Other uses of homologous skin grafts and skin bank bioproducts. Clin Dermatol.

[66] Wulff BC, Parent AE, Meleski MA, DiPietro LA, Schrementi ME, Wilgus TA (2012). Mast cells contribute to scar formation during fetal wound healing. J Investig Dermatol.

[67] Jackson WM, Nesti LJ, Tuan RS (2012). Mesenchymal stem cell therapy for attenuation of scar formation during wound healing. Stem Cell Res Ther.

[68] Williams AR, Suncion VY, McCall F, Guerra D, Mather J, Zambrano JP (2013). Durable scar size reduction due to allogeneic mesenchymal stem cell therapy regulates whole-chamber remodeling. J Am Heart Assoc.

[69] Hur G-Y, Seo D-K, Lee J-W (2014). Contracture of skin graft in human burns: effect of artificial dermis. Burns.

[70] Zeng Y, Zhu L, Han Q, Liu W, Mao X, Li Y (2015). Preformed gelatin microcryogels as injectable cell carriers for enhanced skin wound healing. Acta Biomater.

[71] Huang S, Wu Y, Gao D, Fu X (2015). Paracrine action of mesenchymal stromal cells delivered by microspheres contributes to cutaneous wound healing and prevents scar formation in mice. Cytotherapy.

[72] Li Z, Wang H, Yang B, Sun Y, Huo R (2015). Three-dimensional graphene foams loaded with bone marrow derived mesenchymal stem cells promote skin wound healing with reduced scarring. Mater Sci Eng C.

[73] Zonari A, Martins TM, Paula ACC, Boeloni JN, Novikoff S, Marques AP (2015). Polyhydroxybutyrate-co-hydroxyvalerate structures loaded with adipose stem cells promote skin healing with reduced scarring. Acta Biomater.

[74] Chen S, Shi J, Zhang M, Chen Y, Wang X, Zhang L (2015). Mesenchymal stem cell-laden anti-inflammatory hydrogel enhances diabetic wound healing. Sci Rep.

